# To diagnose primary and secondary squamous cell carcinoma of the thyroid with ultrasound malignancy risk stratification

**DOI:** 10.3389/fendo.2023.1238775

**Published:** 2024-03-01

**Authors:** Xiumei Zhang, Boxiong Wei, Lin Nong, Hong Zhang, Jixin Zhang, Jingming Ye

**Affiliations:** ^1^ Department of Ultrasound, Peking University First Hospital, Beijing, China; ^2^ Department of Pathology, Peking University First Hospital, Beijing, China; ^3^ Department of General Surgery, Peking University First Hospital, Beijing, China

**Keywords:** squamous cell carcinoma (SCC), thyroid, ultrasound, thyroid imaging reporting and data system (TIRADS), fine needle aspiration (FNA)

## Abstract

**Objectives:**

This study aimed to investigate the clinico-ultrasound features of primary squamous cell carcinoma of the thyroid (PSCCT) and secondary SCCT (SSCCT) and evaluate the accuracy of fine needle aspiration (FNA) recommendation for SCCT with American College of Radiology-Thyroid Imaging and Reporting Data System (ACR-TIRADS) and Chinese-TIRADS (C-TIRADS).

**Materials and methods:**

We retrieved 26 SCCT patients (11 PSCCT, 15 SSCCT) from our hospital’s pathology database (5,718 patients with thyroid malignancy) over 23 years. Medical records and ultrasound data of the 26 patients with 27 SCCTs were analyzed retrospectively, and each SCCT focus was categorized based on the two TIRADSs.

**Results:**

For 26 patients (21 males, 5 females) with an age range of 42-81 years, rapidly enlarging thyroid/neck nodules (18/26, 69.2%), dysphagia (7/26, 26.9%), hoarseness (6/26, 23.1%), dyspnea (5/26, 19.6%), cough (4/26, 15.4%), neck pain (2/26, 7.7%), B symptoms (2/26, 7.7%), and blood in sputum (1/26, 3.8%) were presented at diagnosis. Five asymptomatic patients (5/26, 19.2%) were detected by ultrasound. Hoarseness was more common in PSCCT (5/11, 45.5%) than in SSCCT (1/15, 6.7%) (P=0.032). For 27 SCCTs with a mean size of 3.7 ± 1.3 cm, the ultrasound features consisted of solid (25/27, 92.6%) or almost completely solid composition (2/27, 7.4%), hypoechoic (17/27, 63%) and very hypoechoic echogenicity (10/27, 37%), irregular/lobulated margin with extra-thyroidal extension (27/27, 100%), taller-than-wide shape (13/27, 48.1%), punctate echogenic foci (6/27, 22.2%), hypervascularity (23/27, 85.2%) and involved neck lymph (13/26, 50.0%). A total of 27 SCCTs were evaluated as high malignancy risk stratification (≥TR4 and 4B) by the two TIRADSs and recommended FNA in 96.3–100% (26/27, 27/27). Pathologically, more than half of PSCCTs (7/12, 58.3%) and a quarter of SSCCTs (4/15, 26.7%) were poorly differentiated, while moderately and well-differentiated grades were observed in 5 PSCCTs and 11 SSCCTs (P=0.007). Thirteen patients (50.0%) underwent surgery with radical operation in 5 cases (5/13, 38.5%).

**Conclusion:**

SCCT is an extremely rare and aggressive malignancy with a male predominance. PSCCT and SSCCT had similar clinical and ultrasound features except for tumor differentiation and the symptom of hoarseness. SCCT showed a high malignancy risk stratification in ACR-TIRADS and C-TIRADS, with a high rate of FNA recommendation.

## Introduction

Squamous cell carcinoma of the thyroid (SCCT) was previously considered a separate entity but is now classified as a subtype of anaplastic thyroid carcinoma (ATC) due to their analogy (1–3). SCCT comprises primary SCCT (PSCCT) and secondary SCCT (SSCCT, i.e., direct invasion from primary SCC of the adjacent soft tissue and metastasis from a distant SCC) (4). PSCCT with female predominance is most often seen in the seventh decade and more than 60% of the reported cases presented at age 60 or above. Strict adherence to the WHO definition, PSCCT without other cancer components accounts for no more than 0.5% of primary thyroid cancer (5). Thus, pure SCCT is exceptionally rare. SSCCT is about 10 times more common than PSCCT (6, 7). PSCCT is a highly aggressive disease with a poor prognosis with death within the first year in most patients, which is worse than SSCCT (4). Early diagnosis is essential for optimal management and contributes to prompt treatment to prolong life (5, 8).

Ultrasound is an optimal and initial radiological modality to detect and diagnose thyroid nodules (TN) (9). Our previous study, in which 9 PSCCTs in 8 patients were evaluated by 2017 American College of Radiology-Thyroid Imaging and Reporting Data System (ACR-TIRADS) and 2015 American Thyroid Association (ATA) guideline, proposed that PSCCT has certain ultrasonic features for malignancy. The two above guidelines could identify 88.9–100% of PSCCTs as suspicious for malignancy, and the risk stratification of TN could markedly improve diagnostic performance (10).

The 2017 ACR-TIRADS and newly published 2020 Chinese Medical Association proposed the Chinese-TIRADS (C-TIRADS) are widely used for TN evaluation in China (11, 12). We intended to use the two guidelines to assess the risk stratification of SCCT to determine whether it warrants biopsy in clinical practice. So far, the ultrasound features of PSCCT have been described almost exclusively in case reports and small case series. Although SSCCT is more common than PSCCT, its ultrasound findings are exceedingly rarely reported. Therefore, we comparatively analyze the clinic-pathology and ultrasound features of both PSCCT and SSCCT patients to improve the knowledge of SCCT and contribute to the establishment of an optimal diagnostic strategy.

## Materials and methods

### Patient cohort

We retrieved 29 SCCT patients from our hospital’s pathology database of thyroid malignancies (n=5718) from January 1999 to November 2022. The inclusion criteria were as follows: (1) Patients with SCCT proved by pathology; (2) Patients underwent thyroid ultrasound examinations within 3 months of diagnosis. Finally, 26 patients were enrolled due to 3 patients without complete ultrasound data. There were no other primary sites in 11 patients i.e. PSCCT. Other primary sites were identified in 15 patients i.e. SSCCT. The primary sites of SSCCT were esophagus (5/15, 33%), larynx (7/15, 46. 7%), lung (2/15, 13.3%), and cervix uteri (1/15, 6.7%). One of them simultaneously developed SCC in lung and thyroid.

The study complied with the Declaration of Helsinki and was approved by the Ethics Committee of Peking University First Hospital. Individual informed consent was waived for its retrospective nature.

### Thyroid ultrasound examination, 2017 ACR-TIRADS and 2020 C-TIRADS analysis

Thyroid ultrasound examinations were performed with high-resolution ultrasound instruments (GE Logiq 9, GE Logiq E9, Philips HDI 5000, Philips IU22, Philips EPIQ 7, Siemens Acuson S2000 ABVS, Aloka Prosound F75, Esaote Mylab90) equipped with 6-15 MHz linear transducers. Static images were stored in a picture archiving and communication system. All thyroid images were evaluated in a consensus manner by two ultrasound radiologists with more than 10 years of ultrasound experience and uninformed pathological results. The significant ultrasound features of TN were assigned based on ACR-TIRADS and C-TIRADS (**Table 1**). The risk stratification of TN was determined by adding points in the two TIRADSs. Fine-needle aspiration (FNA) was recommended based on the TN risk stratification and its maximum diameter. Extra thyroidal extension (ETE) includes extensive (frank invasion of adjacent soft tissue and/or vascular structures) and minimal ETE (presence of border abutment, contour bulging, or loss of the echogenic thyroid border) (12). In the study, ETE was also adopted to confirm direct invasion of the thyroid from neck mass. Meantime other ultrasound features were analyzed. Enlargement of the thyroid was identified as the anteroposterior diameter of the thyroid >2 cm. Any neck lymph node with two or more of the below features or hypoechoic and enhanced posterior echo was considered suspicious for metastasis: a globular shape or diameter of short axis >5 mm, irregular blurred contours, loss of the normal echogenic hilum, heterogeneity with anechoic/cystic components, Punctate echogenic foci (PEF) and presence of peripheral rather than hilar flow or chaotic vascularity (12–14).

**Table 1:** Assignments of ultrasound features in 2017 ACR-TIRADS and 2020 C-TIRADS.

Ultrasound features	Assignments in ACR-TIRADS (point)	Ultrasound features	Assignments in C-TIRADS (point)
Composition			
Solid/almost completely solid	2	Solid	1
Mixed cystic and solid composition:	1		
Echogenicity			
Very hypoechoic	3	Very hypoechoic	1
Hypoechoic	2		
Hyperechoic/isoechoic	1		
Shape			
Taller-than-wide	3	Taller-than-wide	1
Wider-than-tall	0		
Margin			
Extra thyroidal extension (ETE)	3	Ill-defined/irregular/ETE	1
Irregular/lobulated	2		
Smooth/ill-defined	0		
Echogenic foci			
Punctate echogenic foci (PEF)	3	PEF/suspicious microcalcification	1
Peripheral/rim calcifications	2		
Macrocalcifications	1		
None/large comet-tail artifacts	0	Large comet-tail artifacts	−1

### Statistical analysis

Descriptive data are presented as means and standard deviation (SD) for continuous variables, numbers and percentages (%) for categorical variables. Independent sample t-tests were used for the comparison of continuous data, and Fisher’s exact test for the proportion comparison of categorized data. P<0.05 (two-tails) was considered statistically significant. SPSS 26.0 software (IBM, Armonk, NY, USA) was used for statistical analysis.

## Results

### The patient’s clinical features and medical history

Eleven PSCCT patients (mean age 56.5 ± 10.7 years) included 9 males and 2 females, and 15 SSCCT patients (mean age 62.9 ± 9.0 years) included 12 males and 3 females. The most common presentation was rapidly enlarging neck mass, seen in 69.2% of the patients (18/26). Other symptoms include dysphagia (7/26, 26.9%), hoarseness (6/26, 23.1%), dyspnea (5/26, 19.6%), cough (4/26, 15.4%), neck pain (2/26, 7.7%), B symptoms (2/26, 7.7%), and bloody sputum (1/26, 3.8%) ensued in succession. SCCTs in 5 asymptomatic patients (5/26, 19.2%) were detected by ultrasound examination. Neither bloody sputum nor neck pain was seen in PSCCT patients. Hypocalcemia was found in 1 PSCCT patient (2 cases didn’t have laboratory test). Leukocytosis occurred in 3 PSCCT patients and 6 SSCCT patients (1 case didn’t have laboratory test). During follow-up, PSCCT metastasized to the lung in 27.3% (3/11) and the liver in 9.1% (1/11). In the SSCCT patients, metastasis to the lung, liver, and bone occurred in one patient each (1/15, 6.7%).

History of smoking was found in 8 patients (8/26, 30.8%) with 0.5-2 packs per day (6 current smoker/2 ex-smoker), alcohol consumption with 100-150g per day in 2 patients (2/26, 7.7%), pulmonary tuberculosis in 1 patient (1/26, 3.8%), and diabetes mellitus in 1 patient (1/26, 3.8%). Two PSCCT and 4 SSCCT patients (6/26, 23.1%) had documented or concomitant Hashimoto thyroiditis at diagnosis, with 2 of them with hyperthyroidism (TSH: 0.5uIU/L, 2.36uIU/L; Free T4: 23.82pmol/L, 17.62pmol/L). Hypertension, hyperlipemia, and/or hyperuricemia occurred in 1 PSCCT and 1 SSCCT patients, and chronic obstructive pulmonary disease (COPD) occurred in 2 SSCCT patients. One 81-year-old female with simultaneous lung and thyroid SCC performed radical left mastectomy for invasive ductal carcinoma 18 years ago and vocal cord polypectomy 16 years ago, and had a history of COPD and long-term smoking. One SSCCT patient, secondary to esophagus SCC, had received radiation therapy 2 years ago.

### Diagnostic management and pathological features

Thirteen patients (13/26, 50.0%) with 14 SCCTs underwent ultrasound-guided core needle biopsy (CNB). Among 13 patients who underwent surgery, only 5 (5/13, 38.5%) had radical operation (2 PSCCT, 3 SSCCT patients), 6 (6/13, 46.2%) had palliative/debulking operation (3 PSCCT, 3 SSCCT patients), one had (1/13, 7.7%) incisional biopsy and one had excision operation in another hospital with unavailable surgical data. Two patients underwent tracheotomy due to trachea stenosis resulted from tumor compression and infiltration. Pathologically, 7 PSCCTs (7/12, 58.3%) and 4 SSCCTs (4/15, 26.7%) were poorly differentiated. One well-differentiated PSCCT intermingled with papillary thyroid carcinoma (PTC). Accompanied pathological necrosis was found in 4 SSCCT patients (4/15, 26.7%) and 1 PSCCT patient (1/12, 8.3%). Direct invasion of the thyroid from larynx in 1 SSCCT patient was confirmed by postoperative pathological examination.

### Ultrasound-based risk stratification and recommendations for FNA

Thyroid ultrasound detected 27 SCCTs (12 PSCCTs, 15 SSCCTs) in 26 patients. The mean size was 3.7 ± 1.3 cm (range, 0.9-6.9 cm) with 11.1% (3/27)≤20 mm (1.8 cm and 0.9 cm PSCCTs, 1.0cm SSCCT). Suspicious satellite lesions with less than 5 mm were found in 3 SSCCT patients from laryngeal SCC. 85.2% (23/27) of the thyroid thickness of the disease exceeded 2 cm (2.9 ± 0.9 cm in PSCCT, 2.7 ± 1.1 cm in SSCCT). The normal size of the thyroid lobe of the disease was found in 2 PSCCT patients with 0.9 cm and 1.8 cm tumors respectively, and 1 SSCCT patient with a 2.0 cm tumor abutting the thyroid posterior border. Eight PSCCTs arose in the right lobe, 4 arose in the left lobe and 2 spread to the isthmus. Fourteen SSCCTs evenly arose in the right and left lobe, one 1.0 cm SSCCT from larynx in the isthmus of thyroid. Four SCCTs arose in the setting of nodular goiter, two concurrent with Hashimoto thyroiditis in the real-time ultrasound examination. Two SSCCTs, neck nodular foci abutting thyroid, one from neck lymph node metastasis of cervix uteri, and the other from laryngeal SCC, versus pathology, were not diagnosed by practitioners in the real-time ultrasound reports. The mean size of 5 SCCTs with radical operation was 3.3 ± 1.5 cm (range, 1.1-5.0 cm), while the mean size of 4.5 ± 1.1 cm (range, 3.0-6.0 cm) was found in 7 SCCTs with other surgery.

Twenty-seven SCCTs exhibited solid (25/27, 92.6%) or almost completely solid composition (2/27, 7.4%), hypoechoic (17/27, 63%) and very hypoechoic (10/27, 37%), irregular/lobulated margin (100%). Among the 5 SCCTs with pathological necrosis, only 1 neck nodule of SSCCT abutting and lifting thyroid showed mainly solid mixed with a small central cystic area, while the remaining 4 lesions were hypoechoic solid TNs with various internal hypoechogenicity. Taller-than wide shape was found in 48.1% of SCCTs (13/27) and PEF were found in 22.2% of SCCTs (6/27). Twenty-four SCCTs (24/27, 88.9%) had local ETE in real-time ultrasound reports, and all of them had ETE (5 minimal ETE and 22 Extensive ETE) in TIRADS evaluation. The ultrasound-based ETE showed good agreement with intraoperative observation in 13 patients with surgery (**Table 2**) and showed pathological agreement in 4 of 5 patients with radical operation. Various chaotic internal echoes, such as short linear, multiple sporadic cloudy hyperechoic, central flake attenuation, nodular hypoechoic, or accidental small anechoic area, were observed within the SCCT. Hypervascularity with a high resistive index (RI) was found in 23 SCCTs (85.2%), with a mean RI of 0.85 ± 0.14 (n=17) and a mean peak systolic velocity (PSV) of 23.3 ± 15.4cm/s. 50% of patients (5 PSCCT, 8 SSCCT) were accompanied by neck lymphadenopathy.

**Table 2:** Intraoperative tumor invasion in 13 SCCT patients with surgery.

Gender/age (years)	Surgery	Primary site	Grade	Location	TN size	ETE	Local invasion of intraoperative findings
Male/43	NA	Thyroid	P	Right lobe	3.1cm	Extensive	NA
Female/62	PO	Thyroid	P	Left lobe	3.0cm	Minimal	Esophagus and recurrent laryngeal nerve
Female/53	PO	Thyroid	P	Right lobe + Isthmus	3.9cm	Minimal	Esophagus, recurrent laryngeal nerve, thyroid capsule, multiple lymph node
Male/62	RO	Thyroid	W	Right lobe	5.0cm	Minimal	CCA, thyroid capsule, posterior tissue
Male/60	PO + tracheotomy	Thyroid	P	Left lobe + Isthmus	6.0cm	Both	Esophagus, trachea and muscles
Male/42	RO	Thyroid	M	Left lobe	3.6cm	Both	Surrounding fibrous adipose tissue
Male/58	PO	Esophagus	M	Right lobe	3.7cm	Both	Internal jugular vein, CCA, Trachea and Thyroid capsule, esophagus, Strap muscles
Male/60	RO	Larynx	M	Isthmus	1.1cm	Both	Strap muscles, crico thyroid membrane
Male/48	RO	Larynx	M	Right lobe + Isthmus	2.6cm	Both	Thyroid capsule, posterior muscle, cricoid cartilage
Male/59	PO	Larynx	M	Left lobe + Isthmus	4.7cm	Both	Trachea, thyroid capsule, nerve, esophagus, strap muscles
Male/74	PO + tracheotomy	Esophagus	M	Right lobe	4.5cm	Both	Surrounding muscle tissue, esophagus, trachea
Male/53	RO	Larynx	M	Left lobe	4.2cm	Minimal	Thyroid capsule, trachea
Male/60	Excisional biopsy	Thyroid	P	Left lobe + Isthmus	6.0cm	Extensive	Neck muscles, trachea, posterior soft tissue, esophagus

NA, not available; PO, palliative operation; RO, radical operation; P, poorly differentiation; W, well differentiation; M, moderately differentiation; Both, minimal + extensive; CCA, common carotid artery.

**Figure 1:**
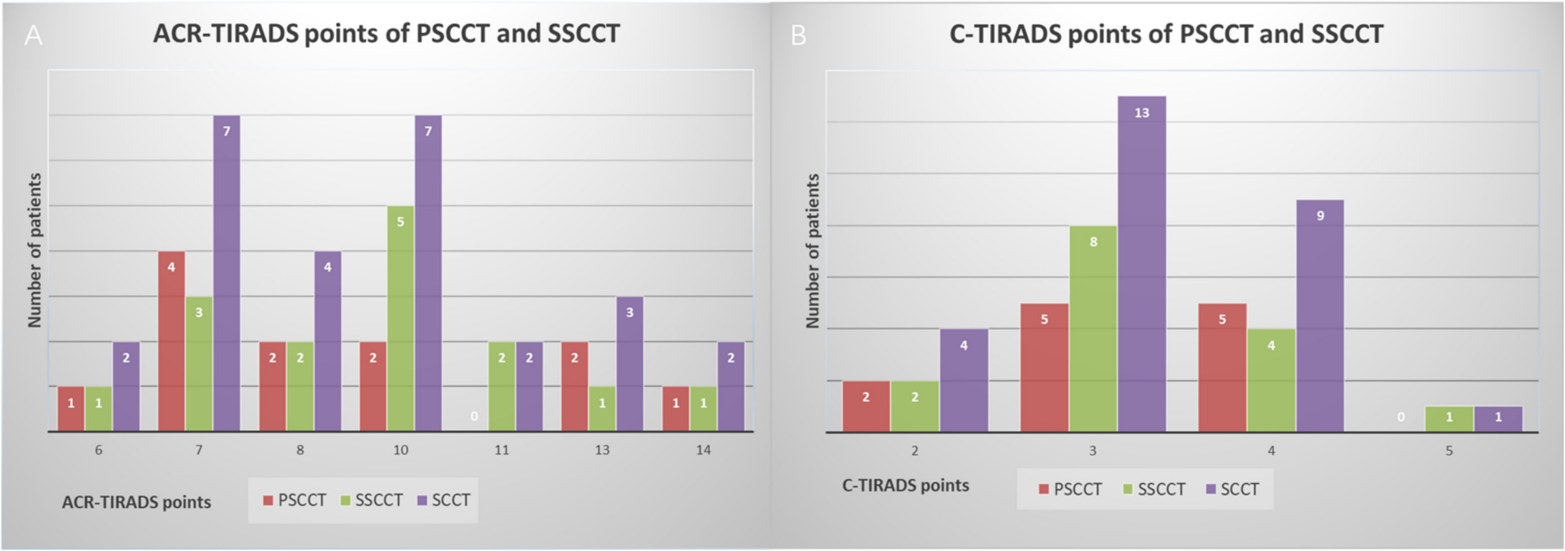
Points of SCCT (PSCCT, SSCCT) based on ultrasound in ACR-TIRADS **(A)** and C-TIRADS **(B)**.

**Figure 2:**
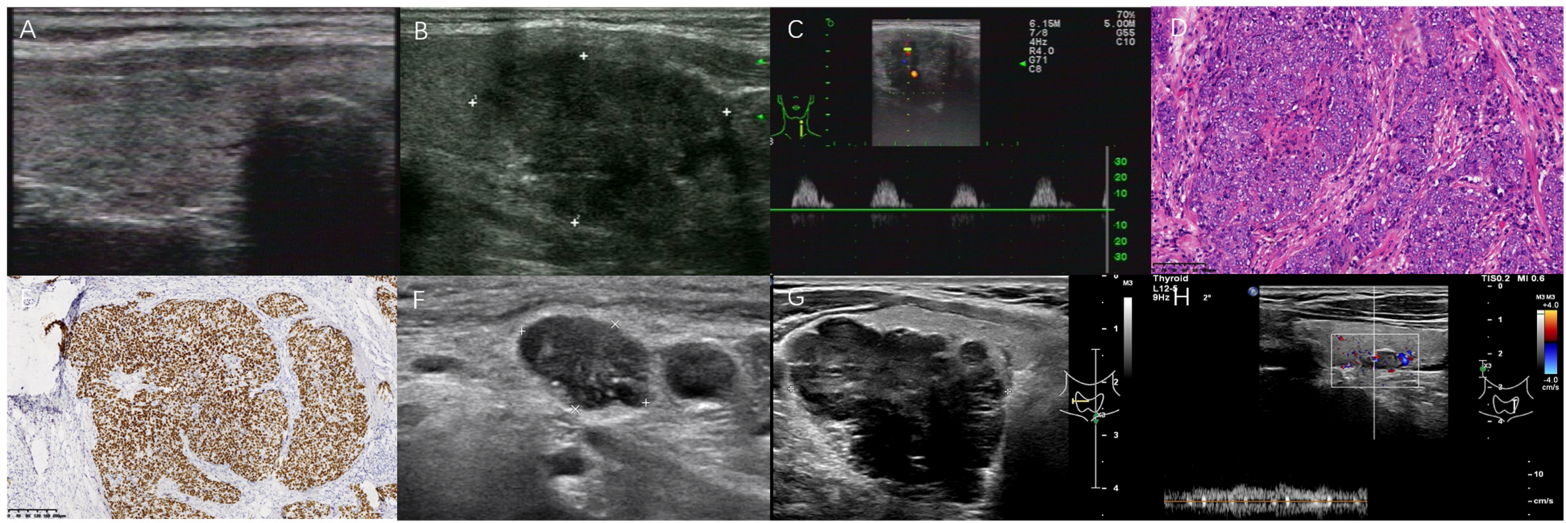
Two poorly differentiated PSCCT patients. A 62-year-old asymptomatic female underwent left lobectomy without any ETE by pathological examination **(A–F)**. Another 52-year-old male with hoarseness underwent CNB **(G, H)**. **(A)** Concomitant Hashimoto thyroiditis with multiple micronodules in right thyroid; **(B)** A 3.1×2.5 cm heterogeneous very hypoechoic TN was evaluated as TR5 with 7 points in 2017 ACR-TIRADS, 4C with 3 points in 2020 C-TIRADS. FNA was recommended; **(C)** Hypervascularity with high RI 1.0, PSV 31 cm/s; **(D)** Tumor cells presented with nest-like infiltration, mainly solid growth (H&E,×100); **(E)** Positive p63 (immunohistochemistry, ×100); **(F)** 1.4×0.9 cm hypoechoic lymph node metastasis with PEF and lobulated margin at 5.5 years after left lobectomy; **(G)** A larger TN with posterior ill-defined margin abutting tissue in right thyroid lobe; **(H)** Contralateral a small 0.9 cm lobulated TN with a central artery, was suggested for FNA recommendation in C-TIRADS, but not in 2017 ACR-TIRADS for its size.

**Figure 3:**
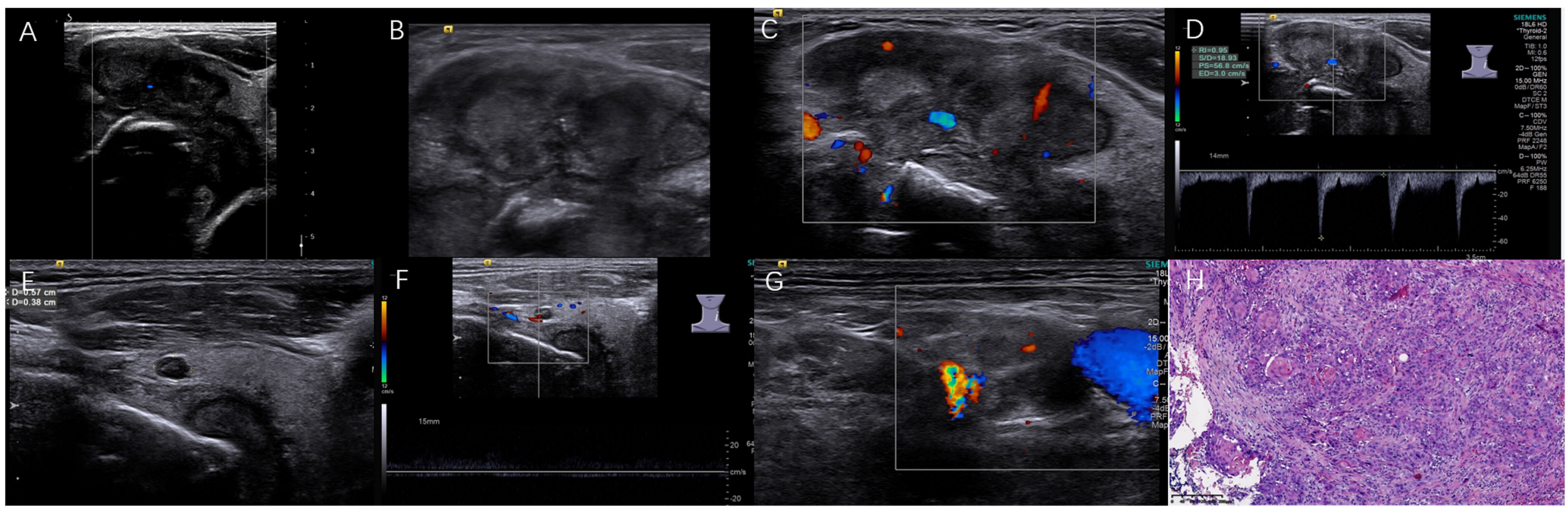
A moderately differentiated SSCCT in a 59-year-old male with the palliative operation and tracheotomy. The irregular solid TN was bestowed 14 points/TR5 in ACR-TIRADS, 5 points/5 in C-TIRADS based on preoperative ultrasound. **(A)** The tumor with extensive ETE involved thyroid left lobe and isthmus; **(B)** PEF within nodular heterogeneous hypoechoic; **(C, D)** Increased blood flow with high RI 0.95 and PSV 56.8 cm/s; **(E, F)** Another adjacent 0.4×0.6 cm small TN with central artery; **(G)** 0.9×1.5 cm neck lymph nodes with ill-defined margin; **(H)** Nodular tumor cells with incidental cornified pearl (H&E, ×100).

Twenty-seven SCCTs were evaluated as TR4 (2/27, 7.4%) and TR5 (25/27, 92.6%), which merited 6-14 points in ACR-TIRADS, and were classified as 4B (3/27, 11.1%), 4C (23/27, 85.2%) and 5 (1/27, 3.7%), which merited 2-5 points in C-TIRADS (**Figure 1**). Neither FNA nor follow-up was recommended for one 0.9cm TR4 nodule since its size in ACR-TIRADS. FNA was recommended for all SCCTs in C-TIRADS. No statistically significant differences in characteristics were found between PSCCT and SSCCT, except for tumor differentiation (P=0.007) and hoarseness (P=0.032) (**Table 3**). **Figure 2** showed ultrasound features of poorly differentiated tumors in 2 PSCCT patients, one with and the other without hoarseness. **Figure 3** revealed a moderately differentiated SSCCT with extensive ETE characterized by frank invasion of the trachea, tracheal cartilage, capsule and strap muscles, and posterior tissue.

**Table 3:** PSCCT and SSCCT patients’ clinic-pathological and ultrasound features at diagnosis.

Patients	PSCCT (*n*=11)	SSCCT (*n*=15)	SCCT(n=26)
Age, Mean ± SD (range), years	56.6 ± 10.7 (42–80)	62.9 ± 9.0 (48–81)	60.2 ± 10.1 (42–81)
Gender			
Male (%)	9 (81.8%)	12 (80.0%)	21 (80.8%)
Female (%)	2 (18.2%)	3 (20.0%)	5 (19.2%)
Chief symptoms^a^			
Rapidly increasing neck mass	8 (72.7%)	10 (66.7%)	18 (69.2%)
Dysphagia	4 (36.4%)	3 (20.0%)	7 (26.9%)
Hoarseness (p=0.032)*	5 (45.5%)	1 (6.7%)	6 (23.1%)
Dyspnea	3 (27.3%)	2 (13.3%)	5 (19.6%)
Cough, stridor and breathy	2 (18.2%)	2 (13.3%)	4 (15.4%)
Neck pain	0	2 (13.3%)	2 (7.7%)
Hemoptysis and blood in sputum	0	1 (6.7%)	1 (3.8%)
Accompanied B symptoms	1 (9.1%)	1 (6.7%)	2 (7.7%)
TNs detected by ultrasound	3 (27.3%)	2 (13.3%)	5 (19.6%)
Medical history			
Hashimoto thyroiditis	2 (18.2%)	4 (26.7%)	6 (23.1%)
Smoking	2 (18.2%)	6 (40.0%)	8 (30.8%)
Pulmonary tuberculosis	0	1 (6.7%)	1 (3.8%)
COPD	0	2 (13.3%)	2 (7.7%)
Alcohol consumption	1 (9.1%)	1 (6.7%)	2 (7.7%)
Invasive ductal carcinoma of left breast	0	1 (6.7%)	1 (3.8%)
DM	0	1 (6.7%)	1 (3.8%)
Hypertension/Hyperlipemia/or hyperuricemia	1 (9.1%)	1 (6.7%)	2 (7.7%)
Tumor metastasis			
Neck lymph node	5 (45.5%)	8 (53.3%)	13 (50.0%)
Lung	3/11 (27.3%)	1 (6.7%)	4 (15.4%)
Bone	0	1 (6.7%)	1 (3.8%)
Liver	1 (9.1%)	1 (6.7%)	2 (7.7%)
Management at diagnosis	Tumor foci (*n*=12)^b^		
CNB	6 (50%)	12 (80.0%)	18 (66.7%)
Surgery	7 (58.3%)	7 (46.7%)	14 (51.9%)
Palliative/debulking surgery	3 (25%)	3 (20.0%)	6 (22.2%)
Radical operation	2 (16.7%)	3 (20.0%)	5 (18.5%)
Incisional biopsy	1 (8.3%)	0	1 (3.7%)
Not available	1 (8.3%)	0	1 (3.7%)
Pathological features			
Tumor grade (P=0.007)*			
Poorly differentiated	7 (58.3%)	4 (26.7%)	11 (40.7%)
Moderately differentiated	2 (16.7%)	11 (73.3%)	13 (48.1%)
Well differentiated	3 (25.0%)	0	3 (11.1%)
Accompanied necrosis	1 (8.3%)	4 (26.7%)	5 (18.5%)
SCCT concurrent with PTC	1 (1/11, 9.1%)	0	1 (1/26, 3.8%)
Ultrasound features			
Tumor size, Mean ± SD, cm	3.8 ± 1.5	3.5 ± 1.2	3.7 ± 1.3
Tumor size ≤20 mm >20 mm	2 (16.7%) 10 (83.3%)	1 (8.3%) 14 (93.3%)	3 (11.1%) 24 (88.9%)
Distribution of thyroid tumors			
Bilateral lobe	1 (9.1%)	0	1 (3.8%)
Left lobe (extensive to isthmus)	7 (1) (63.6%)	6 (1) (46.7%)	14 (2) (53.9%)
Right lobe (extensive to isthmus)	2 (18.2%)	6 (40.0%)	8 (30.8%)
Composition			
Solid	12 (100.0%)	15 (100.0%)	27 (100.0%)
Almost completely solid	0	2 (13.3%)	2 (7.4%)
Echogenicity			
Hypoechoic	8 (66.7%)	9 (60.0%)	17 (63.0%)
Very hypoechoic	4 (33.3%)	6 (40.0%)	10 (37.0%)
Shape			
Taller-than-wide	5 (41.7%)	8 (53.3%)	13 (48.1%)
Wider-than-tall	7 (58.3%)	7 (46.7%)	14 (51.9%)
Margin			
Irregular/spiculated/lobulated	6 (50%)	10 (66.7%)	16 (59.2%)
Extra thyroidal extension	12 (100%)	15(100%)	27 (100%)
Echogenic foci			
No calcification	10 (83.3%)	11 (73.3%)	21 (77.8%)
PEF	2 (16.7%)	4 (26.7%)	6 (22.2%)
Posterior echoes			
Enhancement	3 (25.0%)	5 (33.3%)	8 (29.6%)
No change	4 (33.3%)	7 (46.7%)	11 (40.7%)
Attenuation	3 (25.0%)	1 (6.7%)	4 (14.8%)
Mixed	2 (16.7%)	2 (13.3%)	4 (14.8%)
Intranodular vascularity			
Absent	2 (16.7%)	2 (13.3%)	4 (14.8%)
Increased	10 (83.3%)	13 (86.7%)	23 (85.2%)
Pulsed wave imaging	(n=8)	(n=9)	(n=17)^c^
RI, mean ± SD	0.8 ± 0.2	0.8 ± 0.1	0.8 ± 0.1
PSV, mean ± SD, cm/s	18.4 ± 7.3	27.7 ± 19.5	23.3 ± 15.4
ACR-TIRADS			
Points, Mean ± SD (Median, Range)	9.2 ± 2.8 (8, 6–14)	9.5 ± 2.3 10, 6–14)	9.3 ± 2.5 (10, 6–14)
Risk levels			
TR4 for 5–20%	1 (8.3%)	1 (6.7%)	2 (7.4%)
TR5 for at least 20%	11 (91.7%)	14 (93.3%)	25 (92.6%)
Recommendation by ACR-TIRADS (%)			
FNA	11 (91.7%)	15 (100%)	26 (96.3%)
No follow-up	1 (8.3%)	0	1 (3.7%)
C-TIRADS			
Points, Mean ± SD (Median, Range)	3.3 ± 0.8 (3, 2–4)	3.3 ± 0.8 (3, 2–5)	3.3 ± 0.8 (3, 2–5)
Category for malignancy risk rate			
4B for 10–50%	2 (16.7%)	1 (6.7%)	3 (11.1%)
4C for 50–90%	10 (83.3%)	13 (86.7%)	23 (85.2%)
5 >90%	0	1 (6.7%)	1 (3.7%)
Recommendation by C-TIRADS (%)			
FNA	12 (100%)	15 (100%)	27 (100%)

a Various symptoms may occur in one patient, so the total exceeded 26.

b Two TNs respectively arose in the left and right thyroid glands in one PSCCT patient, so the total PSCCT foci was 12.

c Pulsed wave imaging was performed in 8 PSCCT and 9 SSCCT patients, so the total number was 17.

*P values (<0.05) of the difference were statistically significant.

## Discussion

According to our statistical result, PSCCT was more poorly differentiated (58.3%) than SSCCT (26.7%). PSCCT comprised 0.2% (11/5718) of thyroid malignancy over 23 years, and SSCCT comprised 0.3% (18/5718), which was consistent with the proportions reported in the literature (0.12–0.5%) (5, 10, 15–17). PSCCT is rarer than SSCCT in most literature, and occasionally opposite report which included 17 PSCCT patients and 6 SSCCT patients (18). The rarity of SCCT may be due to the absence of squamous epithelial tissue in the thyroid gland and the rich thyroid arterial supply with fast flow, which prevents the deposition of metastatic cells of SSCCT (19, 20). The origin of PSCCT is far from conclusive (21). More widely accepted hypothesis theories in terms of the origin are that PSCCT results from squamous metaplasia/differentiation of other thyroid diseases, such as nodular goiter, Hashimoto thyroiditis, tumors (papillary, follicular, or medullary cancer), and residual squamous epithelial tissue from the thyroglossal duct or ultimobranchial body during embryonic development (15, 16, 22–24). However, the above clinical conditions only occupy a small proportion of PSCCT. PTC plus Hashimoto thyroiditis only represented approximately 27.3% (3/11) of PSCCT. PTC with a component of SCC could denote a poor prognosis with a few months’ survival (25, 26). Twenty-six point seven percent of SSCCT (4/15) with a history of Hashimoto thyroiditis is higher than 13.7% (16/117) in the literature (5). Thyroid metastatic tumors are more likely to develop in the context of thyroid diseases than normal thyroid glands (27). In pace with earlier diagnosis and treatment of related thyroid disease, especially PTC, PSCCT in epidemiology has decreased in frequency from 0.5% to 0.1% over four decades (28–30). It may be the evidence to support PSCCT originating from the above hypothesis theories. Hypercalcemia-leukocytosis paraneoplastic syndrome was not found in our study, although it has been reported in PSCCT (31). PSCCT is more common in females, with a female-to-male ratio of 1.2-2.4 to 1 in related English literature (5, 28, 29). However, we found reverse gender predominance, female to male ratio of 1 to 4.5 for PSCCT and 1 to 4 for SSCCT, similar to one Chinese study (18). The male is more affected in SCC of the lung, esophagus, trachea, and larynx, which may be one of the causes of male predominance of SSCCT (32). But the rationality of reverse gender predominance for PSCCT is unable to identify. The reverse gender predominance may be probably associated with geographical and ethnic differences, which is needed to investigate further. SCCT occurs in the sixth or seventh decade elderly, older than the mean age in the fifth decade of conventional PTC (5, 28, 33).

SCCT rapidly grows with accompanying symptoms related to the mass effect of cancer. Dysphagia, hoarseness, dyspnea, and cough are common symptoms of SCCT. Hoarseness was more common in PSCCT than SSCCT because the tumor’s immediate vicinity, compression, and infiltration to the recurrent laryngeal nerve are inclined to cause vocal cord paralysis in PSCCT (18). The clinical presentation of metastatic SSCCT could mainly depend on the extent of the primary tumor, with only 20% of metastatic SSCCT with an enlarging neck mass and the rest being discovered at autopsy (30). For the thyroid itself, SSCCT symptoms resembled those of PSCCT at diagnosis. Rapidly enlarging neck masses along with swelling pain in 2 SSCCT patients may be associated with chronic inflammation (8).

The SCCT can be easy to pin down and be screened out by routine thyroid ultrasound examination, even in those asymptomatic patients. SCCT commonly presents as a large TN/neck mass, with only a tiny minority as multiple nodules in metastatic SSCCT, mainly involving one thyroid lobe. 88.9% of SCCTs (24/27) were >2cm at diagnosis. In some cases, oversized SCCTs extend beyond the thyroid gland, bulging the gland contour and abutting adjacent structures, resulting in 48.1% of SCCTs (13/27) with a taller-than-wide shape on the transverse sonogram. This pattern of taller-than-wide shape is different from that of PTC. However, diagnosis of PSCCT in the early stages is challenging due to its rapid growth, lack of symptoms, and absence of typical imaging findings (34). Small-size SCCTs often come across and are misdiagnosed as benign diseases such as nodular goiter or Hashimoto nodule-like change in the real-time ultrasound report.

The ultrasound features of SCCT include solid or almost completely solid composition, hypoechoic and very hypoechoic echogenicity, PEF, and irregular/lobulated margins, especially ETE, which are highly suspicious for malignancy. Very hypoechoic might reflect the assembling of resembling tumor cells. Heterogeneous echotexture with chaotic and scattered hyperechoic was seen in 60% of PSCCT and 20% of SSCCT, likely representing that the hyperplastic fibrous tissue was squeezed and deformed due to rapid proliferation of tumor cells, occasionally accompanied by necrosis, hemorrhage and more chronic inflammatory cell. Pathological necrosis is not always described as an anechoic/cystic area on the sonogram, as among the 5 SCCTs with pathological necrosis, only one from larynx showed a small central anechoic necrotic area, while the other 4 foci were solid with irregular and vertical stripe decaying bands. PEF, corresponding to the psammomatous calcifications associated with PTC, are considered a malignant sign, particularly in combination with other suspicious features. PEF with 22.2% in SCCT are lower than up to 40–50% in PTC and more common than peripheral/eggshell calcification with low specificity (7, 35–38).

The most common spread route of SCCT is local extension into adjacent structures. Ultrasound allows excellent assessment of the local infiltration. Irregular/ill-defined margin, taller-than-wide shape, and ETE are all presentations of SCCT’s invasive nature. A large proportion of SCCTs (88.9–100%) were evaluated to have local ETE in real-time ultrasound reports and the two TIRADS guidelines, higher than 78.9% (15/19) in the literature (39). About 93% of PSCCT showed extensive ETE with prominent vascular and peri-neural infiltration (5). SSCCT that directly spreads to the thyroid from adjacent structures is often incidentally diagnosed by surgical histopathology (30). The direct thyroid invasion can be clarified based on the analogy of minimal ETE, such as the presence of border abutment and lifting contour of the thyroid in 2 SSCCTs can provide clues for thyroid involvement. Posterior anatomic structures of the thyroid could not be clearly described due to limited penetration of high-frequency probe and great over-size tumor. Therefore, due to SCC’s neurotropism, the ill-defined margin abutting the posterior tissue, along with hoarseness, could be reliable signs involving the recurrent laryngeal nerve, which is in line with intraoperative and pathological findings. Nodal metastases are common in neck lymph nodes, affecting 45.5% of PSCCT (5/11). Distant metastases commonly develop in lymph nodes, lungs, bone, liver, and heart (39). Only lung and liver metastases occurred in this study in 36.4% of patients (4/11), lower than the percentage of 73.7% (14/19) reported in an autopsy study (39).

Neovascularization leads to the progressive and rapid growth of SCCT. Most SCCTs were found to have hypervascularity with chaotic, malformed vessel structures. Even microcarcinomas<1.0 cm have a large central supplying artery with low velocity and resistance. Contrary to preconceived ideas, RI and PSV in SSCCT were higher than in PSCCT. Although color Doppler imaging is not included in the TIRADS guidelines due to unreliable discrimination between benign and malignant nodules (40), it can help distinguish solid tissue from echogenic debris or hemorrhage. The absence of flow within a partially attenuated area of SCCT may indicate pathological necrosis (11, 12, 41).

Both ACR-TIRADS and C-TIRADS evaluated 27 SCCTs as in high risk stratification and recommended for FNA except for a 9.7 mm TR4 in ACR-TIRADS. Definitive diagnosis of SCCT was made by FNA in only 6.7–26% of cases (16, 42, 43), so CNB was used to obtain more abundant specimens for higher accuracy in this study. CNB was performed in the 9.7 mm 4B nodule due to its abutting posterior capsule and multifocality in C-TIRADS. However, in ACR-TIRADS, neither FNA nor follow-up was recommended since the TR4 nodule size threshold of FNA is ≧15 mm, and follow-up is ≧10 mm. Although ACR-TIRADS showed higher diagnostic performance and a lower FNA rate (44), small SCCT may be missed for FNA due to the size thresholds. C-TIRADS recommends FNA for 4B nodules >1 cm and >5 mm with minimal ETE or multifocal nodules, which have smaller size cutoffs than those advocated by ACR-TIRADS (45). Thus C-TIRADS was sensitive to recommending FNA of SCCT<1.5 cm, similar to the 2015 ATA guideline (10). The two TIRADSs can identify 1.5-2 cm SCCT foci that warrant biopsy. C-TIRADS could be used to detect newly small SCCT foci (0.5-1.5cm), and ACR-TIRADS might help reduce the unnecessary biopsies in FNA. Although SCCT is a highly aggressive malignancy, patients underwent radical operation obtained a better prognosis. Early detection and definite diagnosis could significantly increase the proportion of radical operation. The mean size of SCCTs with radical operation was found to be smaller than that of those without radical operation. Rapid treatment initiation could improve the long-term outcomes and prolong survival rates (4, 46). Radical operation with adjuvant chemoradiotherapy may obtain a favorable outcome. More than 24-34 months of survival has been reported in PSCCT patients who underwent surgery and radiation therapy with no recurrence or metastasis, longer than the 3-15 months survival commonly reported (31, 46, 47). A 62-year-old asymptomatic female (**Figure 1**) with minimal ETE (thyroid contour bulging) in ultrasound underwent radical operation plus radiotherapy, without ETE in pathology, and survived for 7 years to date, which is the longest survival in our study. Besides, five SSCCT patients survived for 14-72 months during the follow-up period, which is within 3-186 months in a 25-year review (4, 15). Compared to the classic thyroid cancer (predominately PTC), SCCT mainly represents as larger size and more aggressive nature (more ETE and neck lymph node metastasis). Category 5 nodule was more common in PTC (63.2–90%), 4C was more common in SCCT (85.2%) in C-TIRADS, and no difference was found in ACR-TIRADS. The details were showed in **Table S1** (48–51).

The study has several limitations. Firstly, the agreement of local ETE between ultrasound and pathological examination cannot be confirmed in 22 SCCTs since palliative surgery, incisional biopsy and CNB cannot afford the pathological features of the entire tumor. Secondly, the small sample size and single-center retrospective study may lead to the absence of statistical significance between PSCCT and SSCCT in the majority of clinical and ultrasound features. Therefore, studies with large cohorts of patients and prospective research with multi-center are necessary.

In summary, SCCT is a rare malignancy that commonly presents with a rapidly enlarging neck mass with high aggressiveness. PSCCT and SSCCT have similar clinic-pathological and highly suspicious malignant ultrasound features except for hoarseness and tumor differentiation. Ultrasound is an optimal radiological modality to detect and diagnose SCCT based on the two TIRADS guidelines. Finally, the male predominance of SCCT patients in China requires further evaluation.

## Data availability statement

The raw data supporting the conclusions of this article will be made available by the authors, without undue reservation.

## Author contributions

XZ contributed to the conception and design of this study. Material preparation and data collection were performed by XZ, BW, LN, HZ, JZ, and YJ. Analysis was performed by XZ and BW. The first draft of the manuscript was written by XZ, WB. All authors commented on previous versions of the manuscript and read and approved the final manuscript.
