# Supplementary material for: To diagnose primary and secondary squamous cell carcinoma of the thyroid with ultrasound malignancy risk stratification
Source: Front Endocrinol (Lausanne). 2024 Mar 1;14:1238775. doi: 10.3389/fendo.2023.1238775 (PMC10940438; doi:10.3389/fendo.2023.1238775)
Supplement: Supplementary file 1 [file Table_1.docx]

| Table S1. Comparison of clinic-ultrasound features between PTC and SCCT | | |
| --- | --- | --- |
| Patients | PTC (*n*=144/189) | SCCT(n=26) |
| Incidence of thyroid cancer | 80.0–90.0% | 0.5% |
| Age, Mean ± SD (range), years | 45.6±11.9 (18–76) | 60.2 ± 10.1 (42–81) |
| Gender prodominance | Female | Femal^a^/Male |
| Symptoms |  |  |
| TNs detected by ultrasound | More common* | 19.6% |
| Rapidly increasing neck mass | Less common* | 69.2% |
| Accompanied compressing symptoms | Less common* | 85.0% |
| Neck lymph node metastasis | 20.1–50.0% | 50.0% |
| Tumor size |  |  |
| Mean ± SD, cm | 1.5 ± 1.1/2.1 ± 0.8 | 3.7 ± 1.3 |
| >20 mm | 13.0%^&^ | 88.9% |
| ≤20 mm | 87.0%^&^ | 11.1% |
| ≤10 mm | 49.0%^&^ | 7.4% |
| Multifocal tumors | 10.0–20.0% | Incidental/satellite lesions |
| Composition  Solid and almost completely solid | 70.0–98.4% | 100.0% |
| Variable cystic component | 6.3–30.0% | 7.4% |
| Echogenicity  Hypoechoic | 70.8–91.0% | 63.0% |
| Very hypoechoic | 6.9–26.4% | 37.0% |
| Heterogeneous hyperechoic | 2.1–2.8% | 0 |
| Shape  Taller-than-wide | 33.3–50.3% | 48.1% |
| Wider-than-tall | 58.3–66.7% | 51.9% |
| Margin  Smooth or unclear | 45.1–46.0% | 0 |
| Irregular/spiculated/lobulated | 14.6–41.3% | 59.2% |
| Extra thyroidal extension | 12.7–40.3% | 100.0% |
| Echogenic foci  Microcalcification | 25.0–62.5% | 77.8% |
| PEF | 14.6–16.7% | 22.2% |
| Coarse/rim calcification | 9.5–27.8% | 0 |
| Hypervascularity | 90.0%^&^ | 85.2% |
| ACR-TIRADS risk levels |  |  |
| TR3 for ≤5% | 0.7–1.6% | 0 |
| TR4 for 5–20% | 2.1–15.6% | 7.4% |
| TR5 for at least 20% | 65.3–97.2% | 92.6% |
| C-TIRADS category for malignancy risk rate |  |  |
| 4A for 2–10% | 1.4%^&^ | 0 |
| 4B for 10–50% | 6.9%^&^ | 11.1% |
| 4C for 50–90% | 28.5%^&^ | 85.2% |
| 5 >90% | 63.2–90.0% | 3.7% |
| ^a^ Female prodominance was reported in most of literature.  * Quatitative value of PTC was unavailable.  ^&^ Partial quatitative value of PTC was unavailable. | | |
